# Intensive Multimodal Training to Improve Gait Resistance, Mobility, Balance and Cognitive Function in Persons With Multiple Sclerosis: A Pilot Randomized Controlled Trial

**DOI:** 10.3389/fneur.2018.00800

**Published:** 2018-09-28

**Authors:** Johanna Jonsdottir, Elisa Gervasoni, Thomas Bowman, Rita Bertoni, Eleonora Tavazzi, Marco Rovaris, Davide Cattaneo

**Affiliations:** ^1^LaRiCE, Neurorehabilitation, Fondazione Don Carlo Gnocchi Onlus (IRCCS), Milan, Italy; ^2^Department of Multiple Sclerosis, Fondazione Don Carlo Gnocchi Onlus (IRCCS), Milan, Italy

**Keywords:** multimodal training, gait disorders, mobility limitation, multiple sclerosis, cognitive function, balance disorder

## Abstract

**Introduction:** Persons with multiple sclerosis (MS) have deficits in many aspects of physical and cognitive functioning that can impact on mobility and participation in daily life. The effect of a 4 week intensive multimodal treadmill training on functional mobility, balance, executive functions and participation in persons with MS with moderate to severe disability was investigated.

**Methods:** Thirty eight persons with MS admitted to a rehabilitation center participated in a two arm randomized 2:1 controlled trial. Participants in the experimental group received supervised intensive treadmill training including cognitive and motor dual tasks (DT-group, *N* = 26), 5 sessions per week and a control group received the same amount of supervised strength training (S-group, *N* = 12). The participants were assessed before and after the rehabilitation period with the 2 Minutes Walking Test (2MWT), speed and, static and dynamic balance measures, the Frontal Assessment Battery and the Short Form-12 questionnaire. The main hypothesis was related to the superiority of the treadmill intervention based on a greater proportion of people making a clinically relevant gain (15% increase on 2MWT) in gait resistance following treatment. ANCOVA (Analysis of covariance) models adjusting for baseline measurement of the respective outcome variable, as well as sex and age, were used to evaluate differences in efficacy for all variables. *P* was set at 0.05.

**Results:** Nineteen out of 26 persons in the DT-group made a clinically relevant gain and two out of 12 in the S-Group (*P* = 0.001). The DT-group improved more in gait resistance, speed and mobility (*P* < 0.01). Balance and executive functions instead improved moderately in both groups following training while perception of health remained similar in both groups.

**Conclusion:** A four week multimodal training on treadmill was highly effective in augmenting gait resistance and mobility in moderately to severely affected persons with MS.

## Introduction

Persons with multiple sclerosis (MS) can have various deficits, affecting many aspects of physical and cognitive functioning, frequently leading to low levels of physical activity in daily life which further impacts on mobility difficulties through deconditioning of muscles and reduced cardiorespiratory fitness (1–3). Physical inactivity, is particularly common in persons with MS with moderate to severe walking disability and can be due to many factors, such as, inability to maintain steady gait, balance deficits, inability to adapt to environmental demands, fatigue, as well as, cognitive factors (4). Physical activity, in terms of mobility, requires a balance between various interacting systems, locomotion, balance and the central nervous system (CNS) as the coordinating part, where, in particular, executive functions of the frontal cortex appear to be important for mobility (5). In fact, persons with moderate and severe MS disability often have difficulty walking while simultaneously doing motor or cognitive tasks leading to a higher risk of falls during everyday activities (6).

There are some indications in the literature that aerobic and strengthening exercises can change aspects of physical and cognitive performance of persons with MS, with a potentially bigger benefit associated with supervised exercise training (7–11). In particular it is suggested that high-intensity repetitive task-specific practice may be an effective principle when trying to promote motor recovery in neurological diseases (12). For elderly multimodal interventional strategies have been suggested to promote secure mobility by improving attention, dual task performance and executive functions (13). In spite of various studies suggesting a multimodal approach to rehabilitation to increase its general effect and augment physical activity, the effect of intense mobility rehabilitation paradigms including additive cognitive tasks has not been investigated in the MS population. However, there are preliminary indications that 20 minutes of treadmill training can momentarily influence cognitive aspects such as executive control indicating the importance of investigating the inclusion of these aspects in multimodal approaches to rehabilitation (14). Further, a study investigating mobility training on treadmill and strength training found an overall benefit of training accompanied by brain functional reorganization in the sensory-motor network in response to rehabilitation. Although the brain reorganization was not maintained at follow up evaluation the results do indicate some importance of multimodal mobility rehabilitation (15).

Treadmill walking has several benefits for mobility rehabilitation. First, it is an everyday task, walking, second, it lends itself well to a dual task paradigm where other aspects of mobility, such as equilibrium and cognitive factors can be addressed during walking. Third, even persons with severe walking limitations can train walking at various speeds when on treadmill, holding onto handrails and using safety harnesses that minimizes the possibility of adverse events during training. Further, the treadmill paradigm lends itself well to training with progressive task difficulty, numerous rhythmic repetitions, and importantly it can include an aerobic component to improve cardiorespiratory fitness. All of which should lead to improved submaximal exercise tolerance and endurance, more functional mobility and consequently increased ability to carry out activities of daily living.

The aim of this study was to evaluate the safety, feasibility and preliminary effects of a high-intensity rehabilitative multimodal training protocol carried out on treadmill on walking efficacy, mobility, balance, executive function and health-related quality of life in a sample of persons with moderate to severe MS mobility.

## Methods

A consecutive sample of 46 persons with Multiple Sclerosis (MS) was recruited from the inpatient rehabilitation service of the IRCCS Don Gnocchi Foundation, Milan, Italy, in the period from October 2012 to April 2018. Subjects were included in this study if they met the following inclusion criteria: diagnosis of multiple sclerosis according to McDonald's criteria (16), EDSS score ≤ 7 (free from relapses and steroid treatment for at least 3 months), able to stand 30 s, able to walk at least 10 meters independently or with a walking aid, aged between 18 and 80 years, able to understand and follow instructions, stable neurological conditions, and willingness to participate in the study. Subjects were excluded if they had a cardiac pacemaker, any heart condition that their medical doctor considered risky for intense aerobic activity, any pre-existing conditions that affected walking function, diagnosis of depression or psychotic disorder.

The study was conducted in accordance with the Declaration of Helsinki and was approved by the ethics committee of the Don Gnocchi Foundation. Subjects signed an informed consent form before the beginning of the study (ClinicalTrials.gov Identifier: NCT03271125).

## Experimental procedures

**Figure 1:**
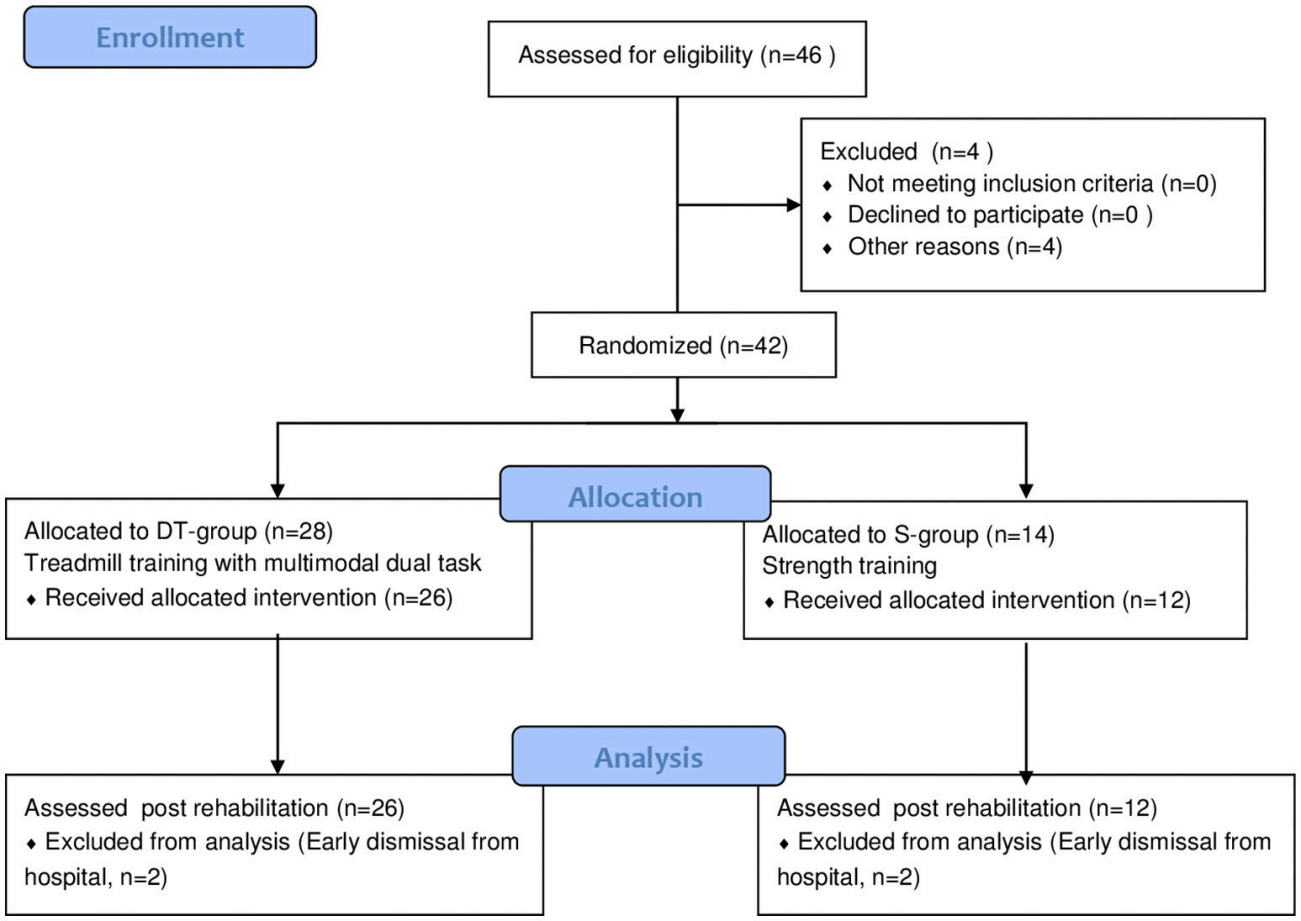
Flow chart of the study.

The study design is a two arm randomized 2:1 controlled trial (see study flow chart in Figure 1). A subgroup of the sample underwent functional magnetic resonance imaging and had a follow up assessment but those results were discussed separately (15).

The participants were assessed before and after the rehabilitation period by researchers blinded to group assignment. Primary outcome measure was the Two Minutes Walking Test (2MWT) (17). Secondary outcome measures were the 10 Meter Walking Test (10MWT) (18), Timed Up and Go (TUG) (19), Berg Balance Scale (BBS) (20), Dynamic Gait Index (DGI) (21), Frontal Assessment Battery (FAB) (22), and the Short Form-12 questionnaire (SF-12) (23).

### Randomization

Participants were consecutively randomized to the experimental (DT-group) or control group (S-Group) in a 2:1 ratio, using a computerized automated algorithm. To ensure a concealed allocation randomization was done after determining eligibility.

### Intervention protocols

Participants in both groups received 15–20 treatments sessions lasting 30 min 5 times per week by experienced physical therapists trained for the study. All participants also followed their usual rehabilitation care protocols as planned. The usual care provided to inpatients in the rehabilitation center consisted in 2 daily rehabilitation sessions focusing on mobility and dexterity to improve function, tailored to individual needs. There were no interferences from the personnel involved in the study as to how the study participants were treated during their usual care rehabilitation, and therapists in the center were not informed as to which group the participants belonged.

#### Treadmill dual task training (DT)

Participants in the experimental group received supervised treadmill training, 4–5 sessions per week. The treatment protocol was aimed at improving participants' resistance, walking velocity, balance and cognitive functions during motor and cognitive (dual) tasks. The treadmill training was carried out without body weight support but if needed the participants were attached to a safety harness. They were also allowed to use the handrails for balance support if needed. Exercise intensity was decided according to the participants scoring of the Rate of Perceived Exertion (RPE) on the Borg Scale (6–20) (24), the training aim was to keep the participants in the 14–16 range of their RPE and treadmill parameters, speed and slope, were regulated based on that. In this way the participants controlled the exercise intensity that was continuously updated based on their perceived effort. Throughout the treadmill session participants were monitored for heart rate and saturation. The 30 min training session consisted of three different walking bouts:

1. An aerobic phase (0–12/30 min): preferred walking speed for the first 3 min, to be increased to taxing walking speed and an increased slope until 12 min, aiming at keeping the RPE at about 14–16 and heart rate under 80% of age predicted maximum heart rate;
2. Dual task phase (12–22/30 min): preferred walking speed with dual task activities comprising motor activities (e.g., balance challenging activities, use of arms in solving motor tasks, changes in walking motions (long steps, walking on toes, with knee lifts), purposeful head rotations, walking with closed eyes) and cognitive activities (e.g., talking, solving logistic, and recalling tasks);
3. An aerobic phase 2 (22–30/30 min): increase in walking speed and slope arriving at RPE 14-16, with the last 3 min at preferred speed.

During the 4-week intervention, walking speed and slope was increased according to RPE of the participants with, however, regard for the heart rate that was always kept within 80% maximum age related frequency. For those participants that could not do the whole 30 min initially, walking duration was gradually increased during the training period as tolerated, with short rest periods allowed if absolutely necessary. If perceived exertion went to 17 or more on the Borg, the training intensity was first reduced slightly in order to have the participant continue at a level 14–16, and only if they felt they could not continue was the exercise interrupted.

#### Strength group (S)

Participants in the Strength training group received supervised muscle resistance training, 4–5 sessions per week. The exercises were specifically aimed at improving strength in muscles involved in walking (hip abductors, quadriceps, plantar flexors, dorsal flexors) and were progressed according to current guidelines from the American College of Sports Medicine (25). Given these recommendations three sets of 10 repetitions were performed bilaterally with appropriate weights for each exercise. Exercise progression was set by increasing the resistance of therabands and/or weights used in the exercises once the participants were able to perform more than the three sets imposed (26).

### Outcome measures

The primary outcome was gait resistance assessed with the 2 Minutes Walking Test (2MWT) (17). The subjects were instructed to walk at their usual speed while the distance they covered in 2 min was recorded in meters. A change of 15% in meters walked from baseline measures to post intervention was considered a clinically important improvement and subjects achieving this threshold were termed responders while those not achieving 15% change were considered non responders.

All other outcomes were considered secondary. The 10 meters timed test was used to test gait speed and the Timed Up and Go (TUG) to assess functional mobility. In the 10 meters timed test the subjects were instructed to walk 10 meters at their comfortable speed, parting from a 0 meters and arriving at 10 meters. The time taken to walk from 2 meters to 8 meters was taken (27). In the TUG the subjects had to stand up from a chair (without armrests), walk 3 m, turn back, and sit down again while being timed. Time taken to complete the test has been shown to be correlated with levels of functional mobility and with scores on clinical static and dynamic balance tests for persons with MS (28, 29).

Static balance performance was assessed with the Berg Balance Scale (BBS), a 14-item scale widely used to assess balance disorders in persons with MS. BBS provides information about patient's balance-related abilities rating performance from 0 (worse) to 4 (best) on 14 items with a maximum total score of 56 (28). Dynamic balance was assessed with the Dynamic Gait Index (DGI), The eight tasks of this scale include walking, walking with head turns, pivoting, walking over objects, walking around objects, and going upstairs. The maximum score is 24, indicating good dynamic balance (28).

Executive function was assessed with the Frontal Assessment Battery (FAB), a short cognitive and behavioral six-subtest battery used for bedside screening of global executive dysfunction. Total score is a maximum of 18, with higher scores indicating better performance. The six subtests of the FAB explore various functions of the frontal lobes, including similarities, mental flexibility, motor series, sensitivity to interference, inhibitory control, and environmental autonomy. The FAB has been used in studies of persons with MS as a measure of executive function and has been validated for the Italian population (30).

Quality of life was assessed with the Short Form-12 questionnaire (SF-12), a shorter version of the commonly used Medical Outcomes Study SF-36. The SF12 is comprised of two domains, physical and mental and gives two composite scores that reflect the perceived health of the participant. Both scores range from 0 and 100, with a higher score indicating better health. Healthy subjects' score is 50 or above in both domains. These SF12-based summaries have been shown to reproduce accurately both scores derived from the full SF36 (23).

### Statistical analysis

Descriptive statistics for the two treatment arms are reported as means and standard deviations, counts and percentages. A primary endpoint was defined as an improvement on the 2MWT of 15% of baseline value or more; participants were accordingly categorized as improved or not improved. Results are reported as differences in proportions with 95% confidence intervals. The presence of outliers was verified and the normality of distributions and the homogeneity of variances were assessed by Shapiro–Wilks and Levene tests. BBS cubed scores and TUG logarithm scores were computed to improve the normality of distribution. Parametric tests and non-parametric tests were used when appropriate.

A primary statistical analysis for all outcomes between the two treatment arms was done using ANCOVA (Analysis of covariance) models with a between group factor (DT-group vs. S-Group) adjusting for baseline measurement of the respective outcome variable, as well as, sex, age, and EDSS. ANCOVA's were computed for the primary outcome (2MWT) and for each of the secondary outcomes. An ANCOVA was also run on the primary outcome including drop outs with an intention to treat approach to verify if the inclusion of drop out subjects would change the results.

Effect sizes expressed as Cohen's d were calculated for the primary outcomes with associated 95% confidence intervals, Cohen's conventions for effect sizes (0.2 small, 0.5 moderate, 0.8 large) were used. Greater improvements in outcomes in the intervention group compared to the control group resulted in positive effect sizes.

All analyses were performed using Statistica software, the *p*-level was set at 0.05 and tests were two-tailed.

## Results

The flow of participants in the study is shown in Figure 1. Of the 42 participants that started the study and were randomized 38 finished the programs, 26 in the DT-group and 12 in the S-group (Figure 1). The reason for not completing the program was early discharge in all four cases, for reasons not related to the study protocol. On average, subjects in both groups completed 18 sessions. There were no adverse events reported during or following either treatment except muscle and general fatigue that was resolved in a couple of hours after the session. After the first couple of sessions some participants complained of muscle soreness and increased stiffness but once ascertained that they were natural consequences of beginning a new intensive motor activity they continued the training without further complaints.

Baseline demographic and disability characteristics of the two groups are shown in Table 1. Baseline clinical characteristics and raw changes after intervention are shown in Table 2. There were no differences between groups in any demographic, disability nor clinical parameters at baseline. The same was true when dropouts were included in the analysis (data not shown).

**Table 1:** Demographic and clinical features of experimental and control groups.

**Variable**	**DT-group (*****N*** = **26)**		**S-group (*****N*** = **12)**		
	***n***	**Mean ± SD**	***n***	**Mean ± SD**	***P*-value**
Age (years)	26	51.4 ± 10.7	12	56.7 ± 5.7	0.07
Disease duration (years)	26	16.3 ± 7.1	12	21.4 ± 10.0	0.11
EDSS	26	5.5 ± 1.1	12	5.6 ± 0.7	0.88
EDSS range		3.5–7		3.5–7	
	**n**	**%**	**n**	**%**	
Sex					0.75
Female	17	44.7	11	28.9	
Male	9	23.7	1	2.6	
MS Type					0.64
Relapse remitting	22	57.9	7	18.4	
Primary progressive	2	5.3	2	5.3	
Secondary progressive	2	5.3	3	7.9	

*n, number; SD, standard deviation; DT, Treadmill Dual Task Group; S, Strength Group; EDSS, Expanded Disability Status Scale; PP, Primary Progressive; SP, Secondary Progressive; RR, Relapsing Remitting*.

**Table 2:** Clinical raw baseline values and raw change scores following intervention of experimental and control groups.

	**DT-Group (*****N*** = **26)**				**S-Group (*****N*** = **12)**		
**Outcome measures**	***n***	**Mean ± SD**	**Change from baseline** **(%)**	***n***	**Mean ± SD**	**Change from baseline** **(%)**	**P** **Between Groups** **at baseline**
2MWT (m)	26	89.1 ± 35.5	29.9(33.3)	12	84.5 ± 34.7	0.17(0.08)	0.79
TUG (s)	26	16.1 ± 7.8	−2.8(17.3)	9^*^	17.4 ± 13.5	2.1(12.1)	0.98
Gait speed (m/s)	26	0.9 ± 0.3	0.21(23.3)	12	0.7 ± 0.3	0.05(7.1)	0.24
BBS	26	42.9 ± 10.3	4.0(9)	12	44.8 ± 9.4	2.7(6)	0.46
DGI	24	15.2 ± 4.4	2.1(13,8)	10	15 ± 5.22	1.9(12)	0.75
FAB	26	14.8 ± 4	1.4(9.4)	12	16.3 ± 1.7	0.4(2,4)	0.72
SF12_Mental	24	39.3 ± 8	1.8(4)	12	42.0 ± 10.2	3.5(8)	0.56
SF12_Physical	24	33.8 ± 7.4	−2.4(−7%)	12	37.4 ± 11.3	−1.4(−3)	0.77

n, number; SD, standard deviation; DT, Treadmill Dual Task Group; S, Strength Group; EDSS, Expanded Disability Status Scale; BBS, Berg Balance Scale, TUG, Timed Up and Go; 2MWT, 2 minutes walking test; DGI, Dynamic Gait Index; SF12_Mental, Short Form-12_ Mental Health Domain; SF12_Physical, Short Form-12_ Physical Health Domain;

* *Timed Up and Go tests of 3 persons missing*.

### Primary outcome

#### Number of persons improved in gait resistance vs. not improved

The results of the primary endpoint analysis are shown in Table 3. Following intervention 19 out of 26 participants (73%) in the DT-group improved clinically in gait resistance (>15%) whereas 2 out of 12 participants (17%) improved in the S-group. The chi-square statistic was 10.5678, *p* = 0.001. The 56% difference in proportions of improved persons in the two treatment arms was significant (*p* = 0.001, CI: 22.4; 74.2).

**Table 3:** Number of improved participants with MS (2MWT Percentage improved ≥15%) at post-treatment evaluation in the DT-group and the S-group, with difference and confidence intervals.

	**DT-Group** ** (*N* = 26)**	**S-Group** ** (*N* = 12)**	***p*-value**	**Difference in proportions**	**95%** ** CI**
**2MWT** ≥**15%**	**N Improved** ** (%)**	**N improved** ** (%)**		**%**	
	19 (73%)	2 (17%)	0.001	56	22.4; 74.2

*CI, confidence interval; DT-Group, Dual-task Treadmill training; S-Group, Strength training; N improved, number improved; 2MWT, 2 Minute Walking test*.

#### All outcomes

Results following intervention for all outcome measures are shown in Table 4. The DT-group improved significantly more (*p* < 0.001) than the S-group in gait resistance, with a 29.9 meters (33.3%) increase in meters walked during the 2MWT while the S-group did not change (0.2 meters, change of 0.3%). The mean difference in change between the two groups was 28.3 ± 7.5 meters and was statistically significant (*p* < 0.001) with a high effect size in favor of the DT-intervention (Cohen's *d* = 1.31, C.I. 0.5–2.0). When dropouts were added to the analysis with an intention to treat approach there was no change in main effects (*p* < 0.001).

**Table 4:** Outcomes of the ANCOVA (adjusted for baseline values, age, and gender) in the DT-group and S-group post intervention and effect sizes with confidence intervals.

**Outcome measures**	***n***	**DT-group** ** Mean Post ± SD**	***n***	**S-group** ** Mean Post ± SD**	Between group differences (DT-S)^*^ Mean (±95% CI)	***p***	**Cohen's *d***
							**Mean (±95%CI)**
**PRIMARY**							
2MWT (m)^ʎ^	26	116.2 ± 21.5	12	87.9 ± 21.5	28.3 (13.04 to 43.60)	0.0006^ƨ^	−1.31(−2.06 to −0.57)
**SECONDARY**							
Gait Speed (m/s)^ʎ^	26	1.0 ± 0.2	12	0.9 ± 0.2	0.2 (0.04 to 0.30)	0.01^ƨ^	−0.5 (-1.19 to 0.19)
TUG (s) ^Ϯ^ ^¥^	26	11.9 ± 2.3	9	14.8 ± 2.9	−2.83 (−0.9 to −4.7)	0.009^ƨ^	1.06 (0.26 to 1.85)
BBS (points) ^Ϯ^^ʎ^	26	48.6 ± 3.7	12	47.4 ± 3.8	1.1(−1.4 to 3.7)	0.39	−0.30 (−0.98 to 0.38)
DGI^ʎ^	24	17.3 ± 2.7	10	17.2 ± 2.7	0.2 (−1.95 to 2.27)	0.87	−0.03(−0.77 to 0.70)
FAB^ʎ^	26	16.8 ± 1.8	12	16.2 ± 1.8	0.6 (−0.07 to 1.84)	0.37	−0.33 (−1.0 to 0.35)
SF12_Mental^ʎ^	24	41.6 ± 8.9	12	44.7 ± 8.8	−3.0 (−9.43 to 3.38)	0.34	0.34 (−0.39 to 1.09)
SF12_Physical^ʎ^	24	35.4 ± 5.3	12	33.6 ± 5.3	1.8 (−2.08 to 5.59)	0.36	−0.34 (−1.08 to 0.40)

n, number; SD, standard deviation; DT, Treadmill Dual Task Group; S, Strength Group; 2MWT, Two-minutes walking test; TUG, Timed up and Go; BBS, Berg Balance Scale; DGI, Dynamic Gait Index; FAB, Frontal Assessment Battery; SF12_Mental, Short Form-12_ Mental Health Questionnaire; SF12_Physical, Short Form-12_ Physical Health Questionnaire.

* Adjusted for pretreatment score, age and gender (T0) by analysis of covariance;

Ϯ These variables did not meet assumptions of data normality and/or homogeneity of variances. In this cases, statistical tests and Cohen d computation were performed on transformed data. Reported between-group differences were estimated from back-transformed results to facilitate interpretation;

ʎ Higher scores indicate better performance;

ƨ P < 0.05 (DT vs. S);

¥ *Lower scores indicate better performance*.

A significant difference was found between groups in improvement in gait speed and mobility. The DT-group improved significantly more on the TUG (*p* < 0.01) than the S-group, with a reduced time to complete the test of 2.8 s (19.1% improvement) vs. an increased time taken by the S-group of 2.9 s (11% worsening). The DT-group also increased their speed significantly more than the S-group (*P* = 0.01), with a 0.2 m/s increase (21.4%) in gait velocity from baseline vs. no change in the S-group, 0.02 m/s (2.5%). Effect sizes were high and significant for both gait speed and [0.95 (0.2–1.7)] and for TUG {−1.06 [−1.8–(−0.2)]} in favor of the dual task treadmill treatment.

There were no other significant differences between groups in improvement at post. Both groups improved their static balance, the DT-group by 4 points on the BBS (9.3%) and the S-group by 2.9 points (6.6%), as well as, dynamic balance with the DT- group improving by 2.4 points on the DGI (15.2%) and the S-group by 1.7 points (10.4%).

Regarding executive function measured with the FAB, there were no differences between groups at baseline nor following intervention. The DT-group with a baseline of mean 14.8 points out of 18 possible increased 1.7 points (11%), while the S-group with a baseline of 15.9 points increased 0.4 points (2.5%). There was a strong ceiling effect on the FAB. Only 10 out of the 26 persons in the DT group had an abnormal scores on the FAB (<15) at baseline and 4 out of 12 in the S-group. Following intervention the persons with abnormal scores in the DT-Group had augmented their scores on the average by 4.2 points and in the S-group by 2.5 points indicating a clinically important difference in executive function following both training modes in the 14 participants with abnormal scores at baseline.

Perception of health was moderately low at baseline in both groups at 39.3 and 42.0 respectively in the DT-group and the S-group on the SF-12_Mental and there was no difference between groups following intervention. The same was true for SF-12-Physical, the groups scored 33.8 and 37.4 respectively at baseline and there was no significant difference within or between groups following reatment.

## Discussion

The present study examined the response of moderately to severely affected persons with MS to an intensive multimodal training consisting in a dual task paradigm with an aerobic component on treadmill (DT-group) and compared it to a group that received a strengthening program of similar intensity (S-group). Following the interventions participants in the DT-group had improved much more in all gait and mobility parameters while factors to do with balance and executive function instead improved moderately in both groups and perception of health remained similar in both groups following training.

Both training modes were well tolerated and there were no adverse events in either case.

### Gait resistance

Nineteen persons out of the 26 persons in the DT-group improved clinically (>15% from baseline) on the primary outcome, gait resistance (2MWT), while only two of the 12 persons in the S-group improved clinically. Large effect sizes were found for gait resistance, speed and mobility outcomes for the DT-group confronted with the S-group (Cohen's *d* 0.95–1.31) indicating a high efficacy of the intensive multimodal training paradigm for gait related activities.

Post intervention the DT-group had a statistically and clinically significant improvement on the 2MWT, increasing on average the distance walked by almost 30 meters (33.3%), while there was no change in the S-group. This improvement is well above the 20–25% change reported by most studies reporting on persons with MS doing gait training on the treadmill and overground (31, 32) and well above the MCD (minimal clinical difference) of 9.6 meters established for the 2MWT by Feys et al. (33). Some differences in intensity levels, both in terms of number of weekly sessions and work load may explain the bigger efficacy of the present study's training protocol on gait resistance compared to others. The intensity of our training was higher in terms of workload during the week compared to a previous study by Braendvik et al. in which subjects performed training 3 times per week for 8 weeks for a total of 24 sessions of treadmill or strength training (34). In contrast, Mostert and Kesselring reported training of similar workload during the week (5^*^30 min per week) with benefits for aerobic fitness, fatigue, and an increase in level of physical activity and perception of health demonstrating that higher disabled persons with MS can benefit from a high training intensity in line with what was observed in the present study (35). An added component in the present study may be the tailoring of the difficulty level throughout the study to the perceived effort of the person itself. This way the persons were training at a high intensity level in all of each session, Further, the training activities were more of the interval type and with variable gait activities, factors that have been shown to be effective in improving cardiovascular parameters and so gait resistance (35–37). Altogether, the results give strength to evidence of treadmill training being preferable to strength training if the goal is to improve walking and walking resistance in persons moderately and severely affected by MS (34, 38).

Two month follow up measures were available only for 9 participants in the DT-group and so were not analyzed, however indications are that the results were maintained in those participants.

### Gait velocity and mobility

The increase in gait resistance of the DT group was reflected in significantly better gait speed and mobility as measured by the TUG in that group compared to the S-group. The improvement observed was around 20% in both parameters. Others have observed similar results following treadmill training, Kalron and colleagues observed an overall reduction of 2.3 s on the 10 meter walking test and 2.4 s on the TUG following treadmill training in a group with severe disability (32). Initial gait speed in both groups was characteristic of an unlimited househould walker according to modified walking categories of persons with MS described by Kempen et al. (18). Following the multimodal treadmill training the DT-group had a mean gait speed of 1.1 m/s characterizing them as limited community walkers. This is an important result indicating an increase in participation possibilities following the intervention. Similarly the positive change in TUG moved the DT group from overall being at a potential risk of falls (16.1 s) according to cut off scores established for older adults (39) (Shumway-Cook) and in a severely affected group of persons with MS according to values established by Kalron and colleagues to no longer being at risk of falls and with a TUG characteristic of moderately affected persons with MS (29). The present results thus strengthens proof from the existent literature adding to evidence based knowledge for the decision of optimal treatment approaches to improve gait speed and mobility in persons with moderate to severe disability levels of MS.

### Balance

The sample was moderately affected in their balance with scores at baseline that were on the verge of the cut off for fall risk established by Cattaneo et al. for persons with MS (28). Both groups improved in static balance, by four points (9%) and approximately three points (7%) on the BBS respectively for the DT-group and the S-group Specifically, an improvement near or over the MCID of three points established by Gervasoni et al. (40) showed a small but clinically important overall effectiveness of both rehabilitation approaches. Results were similar for dynamic balance, there was an increase of 2.3 (15%) and 1.6 points (10%) on the DGI for the DT-group and the S-group respectively indicating that treadmill training was minimally effective in improving dynamic balance since SEM for the DGI has been established at 2 points of the scale and MDC at 4.2 (28). Although the training protocol included parameters such as walking without hand support or turning head and walking with closed eyes during the treadmill training only about one third of the time was dedicated to these dual task activities, including both cognitive exercises and motor activities. It is possible that with longer time spent challenging balance during gait the impact could have been bigger. This hypothesis is in line with a recent review by Gunn and colleagues that reported that a high volume of challenging balance exercise program may be needed in order to have greater benefit on balance and therefore potentially a reduction of falls (41).

### Executive function

There was a clinically and statistically significant increase (*p* = 0.002) in total scores on the FAB in the DT group of almost two points (12%) although the difference between groups was not significant. This improvement seen in response to training that included both aerobic and cognitive-motor components is in agreement with Sangelaji et al. reporting that 24 sessions of combined aerobic and balance training resulted in a significant increase in cognition (measured by the Digit Symbol Modality Test) (42) and a review by Kalron reporting that persons with MS participating in active intervention exercise groups improved in cognitive measures (43).

However, half of our sample had baseline values at or near the maximum score of the scale, so we looked closer at those participants that scored at or below the cut off score (15 points) established by Appellonio et al. (30). This resulted in ten participants in the DT-group and four participants in the S-group with cognitive deficit at baseline. In these participants we found an increase of more than four points in the DT-group and two and a half point in the S-group following intervention indicating a benefit of both training modes, although it was bigger in the DT-group. These results are in support of other works in the literature that have found a positive effect of exercise on cognition from both aerobic and strength training paradigms (43–45). The present findings are only indicative though since the persons with executive function deficits where few and the DT training paradigm included also cognitive exercises. While the findings need to be replicated in bigger studies and with more extensive testing of cognitive function they are interesting since it is known that deficits in executive function have a deleterious effect on gait and balance, especially in complex walking tasks (46). An improvement in this parameter along with improved mobility indicates a benefit to the persons' ability to move in complex community environments.

### Perceived health

Regarding quality of life in terms of health perception, there were no significant changes neither in physical nor the mental domain in both our groups. Our findings do not support the results showed by Motl and colleagues of a significant small beneficial effect of aerobic exercise, but not for non- aerobic exercise (e.g., yoga and resistance training) in improving quality of life (47). One explanation could be that since our sample included also inpatients who cannot experience real life at home during their hospital stay they may have had difficulty in perceiving improvements outside of their daily context.

### General conclusions and limitations

The training was supervised and there were no adversities in combination with the intense training. Using the perceived exertion as a way of letting the subjects themselves control the exercise intensity is a dynamic approach to difficulty adaptation taking into account the participants' status and performance. This approach, along with supervision of heart rate, appears to have been a safe and efficient way of training for our participants. In the current study the emphasis was on achieving speed and stressing the aerobic system during two thirds of the treadmill time. Accordingly, our main results showed a big improvement in the DT-group in all the parameters associated with gait resistance, speed and mobility compared to no change in a S-group that did strengthening exercises at a similar exercise intensity. Regarding balance and cognitive components both groups benefited although only the DT-group had a clinically important change. Balance exercises in a dual tasking paradigm were incorporated into the walking time for about one third of the treadmill time, however, for the rest of the time the participants were allowed to hold onto the side rails in order to achieve faster walking speeds. It is possible that with more time spent in multimodal training the balance and cognitive component of the training would have been even more effective. Given the importance of investigating methods to improve cardiovascular health and increase overall physical activity in persons with MS, further studies incorporating multimodal approaches for people with MS are needed.

There are some limitations of the study the most important being the relatively small sample size. Secondly, while the short-term effects of our exercise training study are encouraging, only a portion of the participants had follow up measures and so the carry over effects on physical activity levels in daily life could not be ascertained. The participants were, however, encouraged to think of daily activities that once they were discharged from the clinic could be used to maintain results gained. This could include walking to work timing themselves, or walking the dog doing progressively longer distances. At last, in the present study aerobic multimodal training was compared to strength training with many of the outcomes being more specific to the aerobic multimodal training. The possibility that strength training was more effective in improving non-aerobic parameters such as, muscle strength cannot be excluded.

## Conclusion

A 4 week supervised multimodal training on the treadmill, including cognitive and motor dual tasks, was effective in augmenting gait resistance and mobility in moderately to severely affected persons with MS with modest benefits also on balance and executive function. This underscores the possibility of improving mobility and cardiovascular health also in persons with MS with relatively high disability levels with a potential impact on their physical activity and daily life participation.

## Ethics statement

This study was carried out in accordance with the recommendations of guidelines for physical activity in multiple sclerosis and Comitato Etico di Fondazione Don Carlo Gnocchi. The protocol was approved by the Comitato Etico, Fondazione Don Carlo Gnocchi. All subjects gave written informed consent in accordance with the Declaration of Helsinki.

## Author contributions

JJ conceived of the design of the work, contributed to acquisition of data, analysis and interpretation of the work. JJ drafted the work, reviewed it critically for important intellectual content and gave final approval of the version to be published. EG participated in conception of the design of the work, data acquisition, analysis, and interpretation for the work. EG drafted the work, reviewed it critically for important intellectual content and gave final approval of the version to be published. TB participated in acquisition of data, interpretation of the work, and drafting. RB participated in the design of the work, contributed to acquisition of data and gave a critical review of the final draft. ET conceived of the design of the work, participated in recruitment and evaluation of participants, and critically reviewed the final version to be published. MR participated in recruitment of participants, drafting, and critical review of the final version to be published. DC conceived of the design of the work, contributed to analysis and interpretation of the work, drafted the work and reviewed it critically for important intellectual content and gave final approval of the version to be published.

### Conflict of interest statement

The authors declare that the research was conducted in the absence of any commercial or financial relationships that could be construed as a potential conflict of interest.
